# Comparison of the Transcriptome of the Ovine Mammary Gland in Lactating and Non-lactating Small-Tailed Han Sheep

**DOI:** 10.3389/fgene.2020.00472

**Published:** 2020-05-21

**Authors:** Jiqing Wang, Huitong Zhou, Jon G. H. Hickford, Zhiyun Hao, Jiyuan Shen, Yuzhu Luo, Jiang Hu, Xiu Liu, Shaobin Li

**Affiliations:** ^1^Gansu Key Laboratory of Herbivorous Animal Biotechnology, Faculty of Animal Science and Technology, Gansu Agricultural University, Lanzhou, China; ^2^Gene-Marker Laboratory, Faculty of Agriculture and Life Sciences, Lincoln University, Lincoln, New Zealand

**Keywords:** RNA-Seq, mammary gland, differentially expressed gene (DGE), lactation, non-lactation, Small-Tailed Han sheep

## Abstract

Small-Tailed Han (STH) sheep are known for their high fecundity, but the survival of lambs is compromised and influences the commercial return from farming these sheep, with this being attributed in part to starvation from insufficient milk production by the ewes. In this study, the transcriptome profiles of the mammary gland of lactating and non-lactating STH ewes were investigated using paired-end RNA sequencing (RNA-Seq). An average of 14,447 genes were found to be expressed at peak-lactation in the STH sheep, while 15,146 genes were expressed in non-lactating ewes. A total of 4,003 differentially expressed genes (DEGs) were identified. Gene Ontology (GO) and Kyoto Encyclopedia of Genes and Genomes (KEGG) analyses revealed that the DEGs were associated with a wide range of cellular components, biological processes and metabolic pathways, including binding activities, signaling pathways, cellular structures, and immune responses. The most highly expressed genes at peak-lactation included *CSN2*, *LGB*, *LALBA*, *CSN1S1*, *CSN1S2*, and *CSN3*, and the 10 most highly expressed genes accounted for 61.37% of the total Reads Per Kilobase of transcript, per Million mapped reads (RPKM). The most highly expressed genes in the mammary gland of non-lactating ewes included *IgG*, *THYMB4X*, *EEF1A1*, *IgA*, and *APOE*, and the 10 most highly expressed genes accounted for only 12.97% of the total gene RPKM values. This suggests that the sheep mammary gland undergoes a substantial development in milk protein synthesis infrastructure and promotion of protein transportation during lactation.

## Introduction

The mammary gland is a complex exocrine organ in mammals and during lactation is responsible for producing critical nutrition as milk for young offspring. Despite only accounting for approximately one percent of the global milk production and being ranked fourth by species in 2013^1^, sheep milk is considered by some people to be superior to cow and goat milk. It has a high percentage of milk solids, contains a high nutrient content, more energy, and smaller fat globules (Selvaggi et al., 2014), compared to other milks. Globally, sheep milk has been recognized as an important and healthy substance for human consumption.

It is well known that milk yield and quality is controlled by both genetic and environment factors, so an in-depth knowledge of the biological mechanisms that control mammary gland development and lactation offers an opportunity to improve milk production. In this respect, attention has become focused on the genes that underpin lactation and where and when they are expressed. As a consequence of its sensitivity and ability to characterize and quantify messenger RNA in different tissues with greater repeatability and low false-positive rate (Marioni et al., 2008; Wang et al., 2009), RNA-Seq transcriptome analysis has been used to analyze genetic mechanisms in different physiological states, or to assess production performance. However, to date transcriptome studies of the mammary gland in livestock have mainly been focused on dairy cows (Bionaz et al., 2012; Wickramasinghe et al., 2012; Cui et al., 2014; Seo et al., 2016; Dai et al., 2018) and dairy goats (Lin et al., 2015; Shi et al., 2015; Crisà et al., 2016).

Despite being a closely related species to goats, sheep lactation typically lasts for 5 months with a peak of milk production between weeks 3 and 4. This compares to a typical 10 months of lactation, with a peak between weeks 5 and 10 in goats (Lérias et al., 2014). This taken together with the difference in milk composition between sheep, and cows and goats, suggests there may be differences in the regulatory mechanisms controlling mammary gland development and milk synthesis in the different ruminant species. Accordingly mammary gland development and activity in sheep require its own investigation.

There are some studies that have described the mammary gland transcriptome of sheep, but these studies were carried out either using milk somatic cells (MSCs) as a proxy for the transcriptome of the mammary gland parenchyma (Suárez-Vega et al., 2015, 2016a,b, 2017), or were taken as mammary gland tissue samples, but were collected from late pregnancy and late lactation New Zealand Romney ewes (Paten et al., 2014, 2015). Little is known about the transcriptome in other sheep breeds, or at different stages of lactation.

The Small-Tailed Han sheep is a Chinese non-dairy breed and it is known for its high fecundity. It has an average lambing rate per ewe of 280%, but the poor survival rate of lambs affects the commercial return to Small-Tailed Han farmers. In other sheep breeds it has been reported that multiple-born lambs often appear to face a milk deficit during the first 3 weeks of lactation (Geenty et al., 1985) and starvation as a consequence of insufficient lactation by ewes, can result in up to 41.7% mortality in multiple-born lambs (Hight and Jury, 1970). The milk supply of the ewe may also affect lamb development, pre-weaning growth-rate and the future productive performance of the lamb (Mellor, 1983; Jordan and Mayer, 1989).

The average milk yield (0.645 L/day) of Small-Tailed Han ewes is low when compared to Katahdin (1.38 L/day) and Saint Croix (1.26 L/day) ewes with multiple-born lambs (China National Commission of Animal Genetic Resources, 2011; Burgos-González et al., 2018), thus knowledge of the biological mechanisms that regulate mammary gland development and lactation in multiple-born-lambs sheep breeds such as Small-Tailed Han is of increasing interest to sheep breeders. It is also of economic importance for sheep production in China and globally.

In mammals, mammary gland development involves repeated cycles of cell growth, differentiation and regression, and non-lactation is one of the important stages in the development processes (Knight and Peaker, 1982). When not lactating, ewes undergo physiological changes and become ready for subsequent mating and lactation. The feeding and management of ewes can also affect mammary gland development and accordingly milk production (Drackley, 1999). However, to our knowledge, there were few studies on the expression profiles of mammary gland during non-lactating period using RNA-Seq in animals, with the exception being dairy cows and yak (Li et al., 2016; Dai et al., 2018; Jiangfeng et al., 2018). There is no study of the molecular mechanisms that affect mammary gland development in the non-lactating period in sheep, or comparisons of mammary gland gene expression in non-lactating and lactating ewes. Accordingly in this study, we used RNA-Seq to profile the ovine mammary gland transcriptome in lactating and non-lactating Small-Tailed Han sheep, and to identify differentially expressed genes (DEGs) between the two states. We also analyzed gene ontogeny (GO) enrichment and Kyoto Encyclopedia of Genes and Genomes (KEGG) pathway of DEGs, with the aim of identifying the possible molecular mechanisms underlying mammary gland development in the two periods.

## Materials and Methods

### Animals and Sample Collection

The sheep experiments were carried out according to the regulations for the care and use of experimental animals (Ministry of Science and Technology, China, approval number 2006-398), and approved by the Animal Care Committee at Gansu Agricultural University.

Nine healthy three-year-old Small-Tailed Han sheep, which were all in their fourth parity and carrying triplet lambs, were investigated. These sheep were reared at the Jinzihe Sheep Breeding Company in Tianzhu County, Gansu Province, China. A sample of mammary gland parenchyma was collected by surgical biopsy at peak-lactation (22 days postpartum for all the ewes). A second biopsy sample was collected from the same nine ewes on 25 days after the cessation of lactation (when the ewes were not pregnant). The tissue samples were immediately frozen in liquid nitrogen and then stored at −80°C until RNA extraction could occur.

Total RNA was extracted from the parenchyma using TRIzol reagent (Invitrogen, Carlsbad, CA, United States) and purified using an RNeasy Mini kit (Qiagen, Hilden, Germany). The RNA concentration was determined and the quality of the RNA was examined, using a NanoDrop 8000 spectrophotometer (NanoDrop Technologies LLC, Wilmington, DE, United States) and 1% agarose gel electrophoresis. The integrity of RNA was then assessed using the Agilent 2100 Bioanalyzer (Agilent, Santa Clara, CA, United States). Only samples with an RNA integrity number (RIN) >7, were used for the study.

### RNA Sequencing and Data Analysis

In order to minimize the effect of variation between individual ewes, the extracted RNA was pooled for RNA sequencing, using an approach described by Paten et al. (2014). Briefly, the RNA extracted from three ewes was pooled into a single sample so as to have equal RNA content per sheep. This created three separate assemblies of RNA from lactating ewes and three assemblies of RNA from the same ewes, in the non-lactating period (i.e., six groups in total).

Complementary DNA (cDNA) libraries for the six groups (3× lactating, 3× non-lactating) were generated using a TruSeq RNA Sample Preparation Kit (Illumina, San Diego, CA, United States). Briefly, mRNA was purified from total RNA using poly-T oligo-attached magnetic beads. Fragmentation was then carried out using divalent cations at 94°C in an Illumina proprietary fragmentation buffer. The first strand cDNA was synthesized using random oligonucleotides and SuperScript II reverse transcriptase (Invitrogen, Carlsbad, CA, United States). The second strand cDNA synthesis was subsequently undertaken using DNA polymerase I and RNase H. The remaining overhangs were converted into blunt ends via exonuclease polymerase activities and then the enzymes were removed. After adenylation of the 3′-ends of the DNA fragments, Illumina PE adapter oligonucleotides (Illumina, San Diego, CA, United States) were ligated to prepare for hybridization.

The library of cDNA fragments were purified to select for fragments of the preferred 200 bp in length using the AMPure XP system (Beckman Coulter, Beverly, CA, United States) and then selectively enriched using Illumina PCR Primer Cocktail (Illumina, San Diego, CA, United States) in a 15 cycle PCR reaction. The products of this amplification were purified using the AMPure XP system (Beckman, Beverly, CA, United States) and quantified using the Agilent High Sensitivity DNA assay (Agilent, Santa Clara, CA, United States) and the Agilent 2100 Bioanalyzer (Agilent, Santa Clara, CA, United States). The cDNA libraries obtained were then sequenced using an Illumina HiSeq 2500 sequencer (Illumina, San Diego, CA, United States) by the Shanghai Personal Biotechnology Co., Ltd. (Shanghai, China).

Raw data were obtained in the FASTQ format. They were arranged according to the number of reads, base amount, Q30 (the proportion of read bases whose error rate is less than 0.1%) and Q20 (the proportion of read bases whose error rate is less than 1%). Clean reads were obtained by removing the raw reads that contained the adapters used to create the cDNAs, and other low-quality reads (those with quality scores <Q20). Quality control indexes for the clean reads were calculated, including base content, GC content and sequence base quality. All the following analyses were then based on the clean data.

The reads were mapped against the ovine genome assembly v3.1 from the Ensembl database using Tophat 2.0 and the alignment results were assessed using RSeQC^2^. Gene abundances were normalized by library and gene length using Reads Per Kilobase per Million reads (RPKM) for each annotated gene.

The differentially expressed genes (DEGs) were identified by comparing the expression levels of the samples of the three groups from the peak-lactation period with the samples from the three groups from the non-lactation period using the DESeq R package (Wang et al., 2010). Genes with |fold change| >2.0 and *p*-value <0.05 were considered to be significant DEGs. Principal components analysis was carried out to evaluate general patterns of variation in expression between the non-lactation and peak-lactation periods using the prcomp function within the R-statistical environment (Groth et al., 2013).

### Reverse Transcription-Quantitative PCR

Sixteen of the DEGs identified were selected for reverse transcription-quantitative PCR (RT-qPCR) analysis to corroborate the RNA-Seq results. These included eight genes that were up-regulated during the non-lactating period compared to the peak-lactating period: *WIPF1* (WAS/WASL interacting protein family member 1), *PAPLN* (papilin, proteoglycan-like sulphated glycoprotein), *GDF10* (growth differentiation factor 10), *MYOF* (myoferlin), *FSCN1* (fascin actin-bundling protein 1), *STAT6* (signal transducer and activator of transcription 6), *CD4* (cluster of differentiation 4 glycoprotein) and *MAP3K14* (mitogen-activated protein kinase 14); and eight down-regulated genes [*HSPA9* (heat shock protein family A member 9), *LALBA* (lactalbumin alpha), *STAT5a* (signal transducer and activator of transcription 5a), *JAK1* (Janus kinase 1), *XDH* (xanthine dehydrogenase), *LPL* (lipoprotein lipase), *FAM78B* (family with sequence similarity 78 member B) and *LCN2* (lipocalin 2)]. Two additional DEGs [*IgA* (immunoglobulin A) and *IgG* (immunoglobulin G)] that were highly expressed during the non-lactation period were also selected for analysis by RT-qPCR. The genes *PRPF3* and *CUL1* were chosen as internal references to normalize the mRNA levels of the DEGs, using the approach suggested by Paten et al. (2014).

The RNA samples for RT-qPCR that were the same as those used for the RNA-Seq analysis, were used to synthesize cDNA using SuperScript II reverse transcriptase (Invitrogen, Carlsbad, CA, United States). PCR primers for the above genes were then designed using primer 5.0 (Table 1) and synthesized by the Takara Biotechnology Company Limited (Dalian, China). The RT-qPCR was performed in triplicate using the 2 × ChamQ SYBR qPCR Master Mix (Vazyme, Nanjing, CHN) on an Applied Biosystems QuantStudio 6 Flex (Thermo Lifetech, United States) platform. The relative expression levels of the genes were calculated using a 2^–ΔΔ*Ct*^ method (Livak and Schmittgen, 2001).

**TABLE 1:** PCR primers used for RT-qPCR.

Gene	Forward primer sequence (5′–3′)	Reverse primer sequence (5′–3′)	Expected size of amplicon (bp)	Annealing temperature (°C)	Reference sequence
*LALBA*	TACGGAGGTGTCAGTTTG	TCCTTGAGTGAGGGTTCT	160	60	NM_001009797
*STAT5a*	CAACGGCATGTCTGTGTCCT	AGTGGGGCTTGTGATGTTTCTT	124	60	NM_001009402.2
*HSPA9*	TGAAGACTTTGACCAGGCCT	GAACTCTCTGAAGCGCCATG	100	60	XM_004008840.4
*JAK1*	CTACAATGGCGAGATCCCCT	CTCTGGTTGGGGTCGTAGTT	105	60	XM_015092018.2
*XDH*	AGGGAACATCATCACAGCCA	GTAGCTGGGGAAGAAGGTGT	132	60	XM_027966969.1
*LPL*	TGCCTTACAAAGTCTTCCA	GCCACAGTGCCATACAGA	106	60	XM_027963889.1
*FAM78B*	AGGGAAGGGAGAGTGAAAGC	TTCATGCTGACGGAGAACCT	127	60	XM_004002724.4
*LCN2*	AACGTCACCTCCATCCTGTT	GTGTAGCTCCTTATCCCGGG	127	60	XM_012147566.2
*WIPF1*	CGGAGGTGGTGGAAGTTTTG	GTGAAAGGTTTGGCGGATGT	166	60	XM_027964833.1
*PAPLN*	GAAGCCAATGACCTCAGCAG	GAGGTAGTATTCGCCACGGA	134	60	XM_027971986.1
*GDF10*	CCGGAAGAAGCAATGGGATG	CAGTAGTAGGCGTCGAAGGA	120	60	XM_004021551.4
*MYOF*	CGACCAGAAACCTCCTTCCT	CCACAAAGAGCAGCAGGATC	123	60	XM_012102564.3
*FSCN1*	ACGAAGAGACCGACCAAGAG	CATTGGACGCACGCAGAATA	197	60	XM_027961638.1
*STAT6*	ATCTTCAGTGACAGCAGCCT	CACCATCAAACCACTGCCAA	120	60	XM_027967569.1
*CD4*	ATATGCCGCTCCAGTGCTAT	CAGCCCAGATGACGGATACT	139	60	XM_012113399.2
*MAP3K14*	TCATGGAACTGCTGGAAGGT	CGTTGTCGGCTTTCACATCT	158	60	XM_004012997.3
*IgA*	CTCCTGCTCTGCCACCTACC	GCGTCACCAGCTCGTTGT	137	60	ENSOARG00000008862
*IgG*	CAAGGTCCACAACAAAGGC	GCGATGTAGTCTGGGTAGAAGC	171	60	ENSOARG00000009143
*PRPF3*^1^	ACAGATGATGGAAGCAGCAA	GGTTGGGAGGATGAAGGAGT	101	60	XM_004002450.1
*CUL1*^1^	AAAAATACAACGCCCTGGTG	CTGAGCCATCTTGGTGACTG	116	60	XM_004008343.1

*^1^PRPF3 and CUL1 were chosen as internal reference genes as suggested by Paten et al. (2014).*

### Gene Ontology and KEGG Pathways Analyses

Gene ontology (GO) enrichment analysis^3^ of the DEGs was performed and three major functional ontologies including biological process (BP), molecular function (MF) and cellular component (CC) were annotated for the DEGs. Pathway analyses were used to identify the pathways that contain DEGs using the Kyoto Encyclopedia of Genes and Genomes (KEGG) database^4^. The significant GO terms and pathways (*P* < 0.05) were classified based on Fisher’ exact and χ^2^ tests, and the *P*-values were corrected (*Pcor*-value) using the calculated False Discovery Rate (FDR) value (Wang et al., 2014).

## Results

### Summary of the RNA-Seq Data

Six separate cDNA libraries were constructed from the mammary gland tissue biopsies (RNA from nine samples collected at peak-lactation and pooled into three groups, and RNA from nine samples collected in the non-lactating period, and pooled the same way into another three groups). These were analyzed using an RNA-Seq approach. All the raw reads obtained in the study have been deposited in GenBank with accession numbers SRR11300645–SRR11300650, and the summary statistics of the RNA-Seq data are shown in Table 2. On average, 141,623,156 raw reads were produced from the cDNA libraries constructed from the three groups of sheep at peak-lactation and 140,182,560 raw reads were produced on average for the three groups of non-lactating ewes. After filtering (by removing adaptors and low-quality reads with quality scores <Q20), an average of 140,631,794 and 139,187,264 high quality clean reads were obtained from the three groups of sheep at peak-lactation and those that were no longer lactating, respectively. Of these cleaned reads, 108,370,860 (77.06%) and 107,438,649 (77.19%) mapped well to the ovine genome assembly (Oar_v3.1), with a unique match ratio of 97.12% and 97.08%, respectively. Using a cut-off of >0.01 RPKM to define potentially meaningful expressed genes [see Mortazavi et al. (2008)], we detected an average of 14,447 and 15,146 genes expressed in ovine mammary gland tissues during peak-lactation and in the non-lactating period, respectively, with 13,928 genes being expressed at both stages. A principal component analysis (PCA) analysis revealed that all of the six grouped samples were clustered into two distinct groups, which reflected their status as lactating/non-lactating ewes. The first and second principal components accounted for 51.98 and 42.53% of the total variation, respectively (Supplementary File 1).

**TABLE 2:** The summary of the RNA-Seq data.

Samples	Average raw reads	Q20 (%)	Q30 (%)	Average clean reads	Average mapped reads	Mapped reads (%)	Average unique reads	Unique reads (%)
Peak-lactation	141623156	95.91	90.23	140631794	108370860	77.06	105249779	97.12
Non-lactating	140182560	96.02	90.87	139187264	107438649	77.19	104296069	97.08

*Differentially expressed genes (DEGs) at the peak of lactation and in non-lactating mammary gland parenchyma.*

A total of 4,003 DEGs were identified to be differentially expressed when comparing the non-lactating and peak-lactation periods (Supplementary File 2). Of these, 1,922 (48%) had higher expression in the non-lactating period compared to peak-lactation and are therefore referred to as “up-regulated,” while the remaining 2,081 DEGs (52%) had higher expression at peak-lactation and are accordingly referred to “down-regulated” in this study.

### Gene Ontology (GO) Enrichment Analysis of the DEGs

**FIGURE 1:**
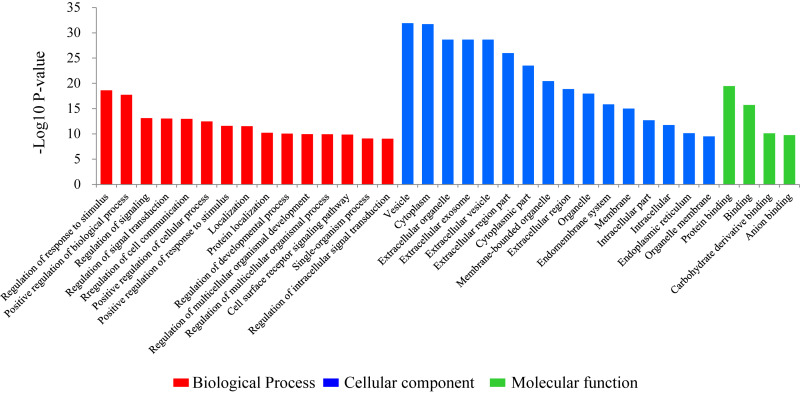
Gene ontology (GO) classification of the differentially expressed genes comparing the non-lactating and peak-lactation periods. The most enriched biological process, cellular component and molecular function GO terms are shown.

To investigate the biological functions of the DEGs, a GO enrichment analysis was performed. The significant GO enrichment terms (*Pcor* < 0.05) for the annotated DEGs were classified into 283 functional groups, across the three GO established categories: “biological processes” (193 terms), “cellular component” (53 terms) and “molecular function” (37 terms). The top 15 significant with the lowest *Pcor*-value were: “vesicle” (*Pcor* = 1.21E-32), “cytoplasm” (*Pcor* = 1.95E-32), “extracellular organelle” (*Pcor* = 2.13E-29), “extracellular exosome” (*Pcor* = 2.13E-29), “extracellular vesicle” (*Pcor* = 2.13E-29), “extracellular region part” (*Pcor* = 1.04E-26), “cytoplasmic part” (*Pcor* = 3.03E-24), “membrane-bounded organelle” (*Pcor* = 3.46E-21), “protein binding” (*Pcor* = 3.42E-20), “extracellular region” (*Pcor* = 1.31E-19), “regulation of response to stimulus” (*Pcor* = 2.37E-19), “organelle” (*Pcor* = 1.05E-18), “positive regulation of biological process” (*Pcor* = 1.83E-18), “endomembrane system” (*Pcor* = 1.42E-16) and “binding” (*Pcor* = 1.97E-16) (Figure 1). Among these 15 significant terms, eleven belonged to the “cellular component” category, two belonged to the “biological processes” category and two belonged to the “molecular function” category.

### KEGG Pathway Analysis of the DEGs

**FIGURE 2:**
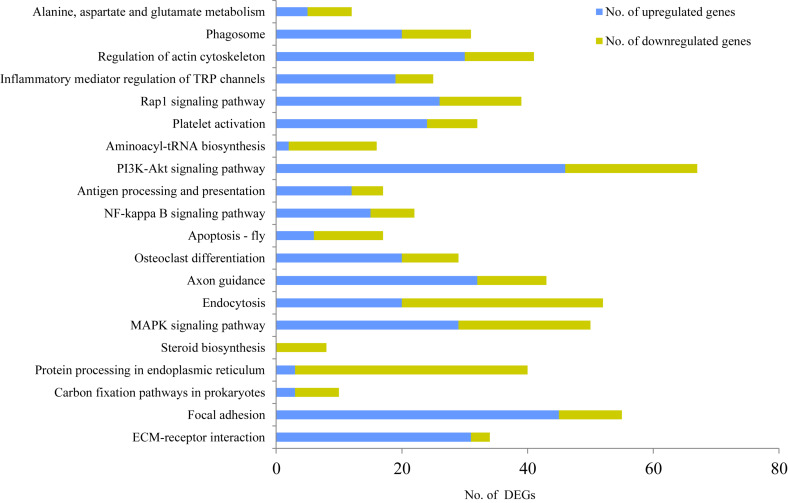
KEGG enrichment analysis for differentially expressed genes (DEGs) between non-lactating and peak-lactation periods. Blue and green bars represent the number of up-regulated and down-regulated DEGs (when comparing non-lactation to peak-lactation), respectively.

To further identify the possible functional pathway of DEGs in the two mammary gland development stages, a KEGG pathway enrichment analysis was performed. The most enriched pathways were extracellular matrix (ECM)-receptor interaction (*P* = 2.80E-06, 69 genes, including 34 DEGs), followed by focal adhesion (*P* = 8.01E-06, 136 genes, including 55 DEGs), carbon fixation pathways in prokaryotes (*P* = 7.39E-05, 13 genes, including 10 DEGs), protein processing in endoplasmic reticulum (*P* < 0.001, 100 genes, including 40 DEGs), steroid biosynthesis (*P* < 0.001, 10 genes, including eight DEGs), mitogen-activated protein kinase (MAPK) signaling pathway (*P* < 0.001, 135 genes, including 50 DEGs), endocytosis (*P* < 0.001, 142 genes, including 52 DEGs), axon guidance (*P* < 0.001, 114 genes, including 43 DEGs), osteoclast differentiation (*P* < 0.001, 70 genes, including 29 DEGs), apoptosis-fly (*P* < 0.001, 34 genes, including 17 DEGs), NF-kappa B signaling pathway (*P* < 0.001, 49 genes, including 22 DEGs), antigen processing and presentation (*P* = 0.0011, 35 genes, including 17 DEGs), PI3K-Akt signaling pathway (*P* = 0.0013, 203 genes, including 67 DEGs), aminoacyl-tRNA biosynthesis (*P* = 0.0016, 33 genes, including 16 DEGs), platelet activation (*P* = 0.0016, 83 genes, including 32 DEGs), Rap1 signaling pathway (*P* = 0.0018, 107 genes, including 39 DEGs), inflammatory mediator regulation of TRP channels (*P* = 0.0032, 63 genes, including 25 DEGs), regulation of actin cytoskeleton (*P* = 0.0033, 117 genes, including 41 DEGs), phagosome (*P* = 0.0041, 84 genes, including 31 DEGs), and alanine, aspartate and glutamate metabolism (*P* = 0.0044, 24 genes, including 12 DEGs) (Figure 2).

Among these pathways, steroid biosynthesis was specific for the peak-lactation samples, whereas there did not appear to be any pathway specific for the non-lactating samples. According to the number of up-regulated genes and down-regulated genes, the KEGG pathways could be categorized into two groups: (1) pathways with the majority of DEGs being up-regulated during non-lactation (e.g., ECM-receptor interaction, focal adhesion, MAPK signaling pathway, axon guidance, osteoclast differentiation, NF-kappa B signaling pathway, antigen processing and presentation, PI3K-Akt signaling pathway, platelet activation, Rap1 signaling pathway, inflammatory mediator regulation of TRP channels, regulation of actin cytoskeleton and phagosome); and (2) pathways with the majority of DEGs being down-regulated during non-lactation (e.g., carbon fixation pathways in prokaryotes, protein processing in endoplasmic reticulum, steroid biosynthesis, endocytosis, apoptosis-fly and aminoacyl-tRNA biosynthesis).

### Validation of RNA-Seq Results Using an RT-qPCR Approach

**FIGURE 3:**
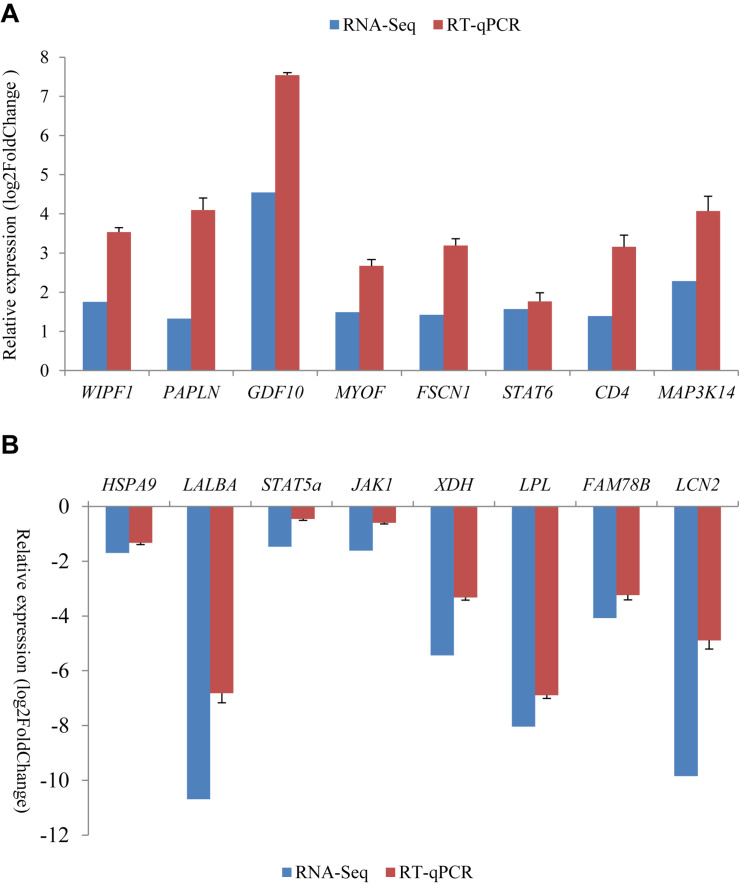
Comparison of gene expression levels obtained by RNA-Seq, with those measured with RT-qPCR for 16 randomly selected differently expression genes (DEGs). These included eight up-regulated genes **(A)** and eight down-regulated genes **(B)** in the non-lactating period compared to peak-lactation. The RT-qPCR results are presented as mean ± SD for three replicates with the SD being shown in the error bars.

For all of the 16 randomly selected DEGs, RT-qPCR generated results with expression patterns that reflected the RNA-Seq results, although fold-change values in the RT-qPCR and RNA-Seq data were variable (Figure 3). This is probably due to the different computational methods used for the different analytical platforms. The pearson correlation coefficients for relative expression levels of these genes in the two periods between qRT-PCR and RNA-seq was 0.963 (*P* = 0.000). Overall, the RT-qPCR results were taken to confirm the reliability and repeatability of the RNA-Seq results.

### The Ten Most Expressed Genes at Peak-Lactation and During the Non-lactating Period

**FIGURE 4:**
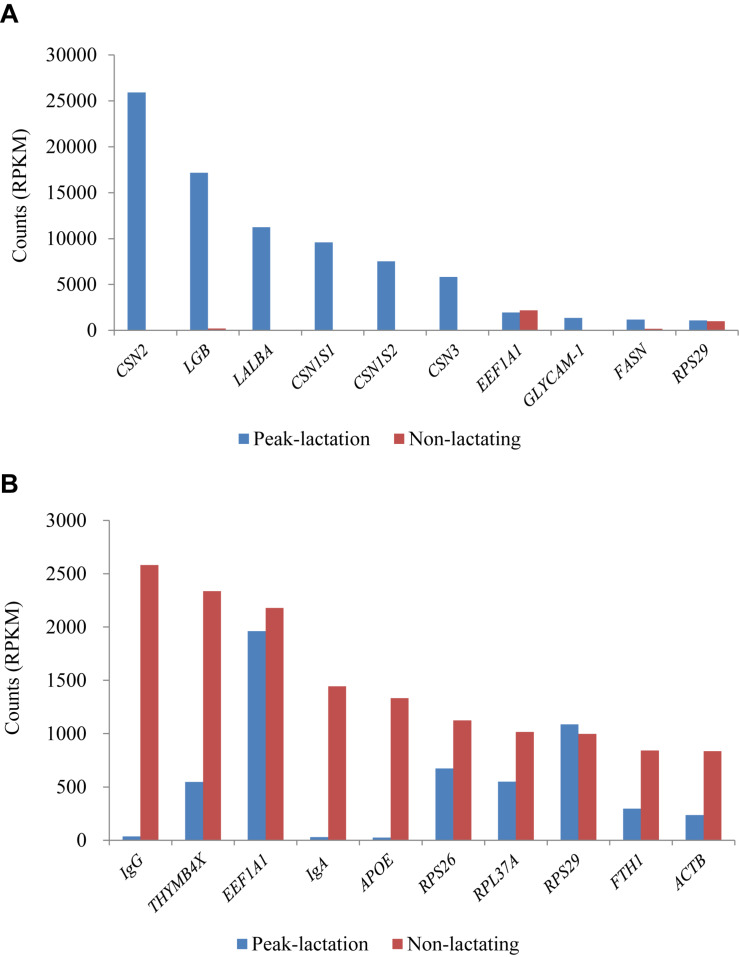
Ten most expressed genes identified in the peak-lactation **(A)** and non-lactating **(B)** periods.

The ten genes with the highest RPKM values (1,086–25,919 RPKM) during the peak-lactation period were *CSN2* (β-casein), *LGB* (β-lactalbumin), *LALBA* (α-lactalbumin), *CSN1S1* (α-S1 casein), *CSN1S2* (α-S2 casein), *CSN3* (κ-casein), *EEF1A1* (eukaryotic translation elongation factor 1 alpha 1), *GLYCAM1* (glycosylation-dependent cell adhesion molecule 1), *FASN* (fatty acid synthase) and *RPS29* (ribosomal protein S29). These ten genes accounted for 61.37% of the total gene RPKM values at peak-lactation. Except for *EEF1A1* and *RPS29*, the other eight genes were found to be at a very low level of expression during the non-lactation period (Figure 4A).

During the non-lactation period, the ten genes with the highest RPKM values (836–2,582 RPKM) were *IgG*, *THYMB4X* (thymosin beta-4, X-linked), *EEF1A1*, *IgA*, *APOE* (apolipoprotein E), *RPS26* (ribosomal protein S26), *RPL37A* (ribosomal protein L37a), *RPS29, FTH1* (ferritin heavy chain 1) and *ACTB* (beta actin) (Figure 4B). These ten genes accounted for 12.97% of the total gene RPKM values in the non-lactation period. Of these, three genes (*IgG*, *IgA*, and *APOE*) were found to be hardly expressed and five genes (*THYMB4X*, *RPS26*, *RPL37A*, *FTH1*, and *ACTB*) showed a significant decrease in the expression level in the peak-lactation period (Figure 4B). The remaining two genes (*EEF1A1* and *RPS29*) were also highly expressed in the lactating period (Figure 4B).

**FIGURE 5:**
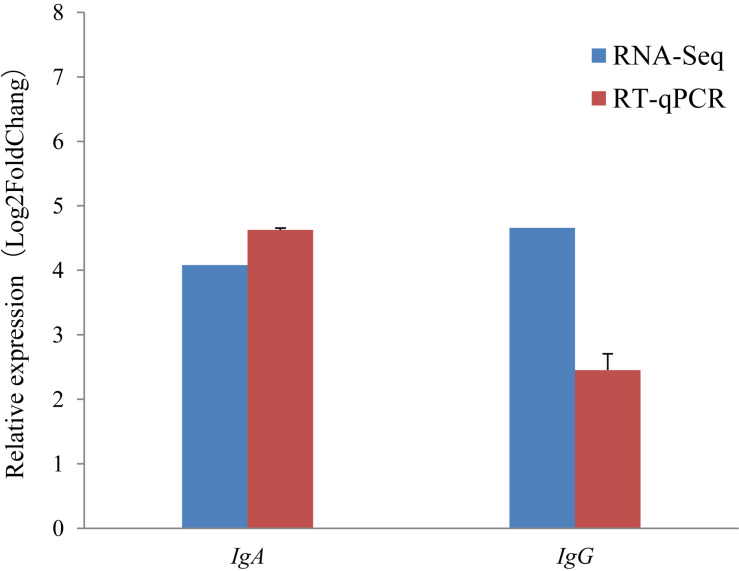
Comparison of gene expression levels obtained with RNA-seq, with those measured with RT-qPCR for *IgA*, and *IgG*. The red colored bars show the mean for three replicates for each sample RT-qPCR and the error bars indicate standard deviation (SD). Fold change is the expression level in the non-lactating period divided by the expression level at peaking-lactation for the genes.

It is notable that the two immunoglobulin genes *IgA* and *IgG* were found to be highly expressed during the non-lactating period, but were hardly expressed during the lactating period using the RNA-Seq approach (Figure 4B). To validate this, the relative expression levels of *IgA* and *IgG* were also investigated using the RT-qPCR approach. The RT-qPCR analyses revealed that these two genes had expression levels that were similar to those measured by RNA-Seq (Figure 5). The pearson correlation coefficients for relative expression levels of *IgA* and *IgG* between qRT-PCR and RNA-seq was 1.000 (*P* < 0.01). These confirm that *IgA* and *IgG* were up-regulated in the non-lactating period, but down-regulated in the lactating period.

## Discussion

This is the first study describing changes in gene expression determined using an RNA-Seq approach in the ovine mammary gland of lactating and non-lactating sheep. The depth of sequencing is an important measure for the RNA-Seq approach and it determines how effectively the method detects transcripts. For abundant and moderately abundant transcripts, 30–40 million reads are considered to be sufficient for detection, while for the low-expression transcripts, a greater sequencing depth is needed to enable accurate detection and to assess their abundances (Mortazavi et al., 2008). In this study the RNA-Seq produced an average of 142 and 140 million reads for the group samples collected at peak-lactation and during the non-lactating period, respectively. This is substantially more than those reported in a previous study of mammary gland tissue with Romney sheep with 21–64 million reads (Paten et al., 2015). This suggests that more transcripts are detected in this study, especially for those occurred at a relatively low-abundance. This argument is supported by a larger number of expressed genes detected in this study compared to the expression of 10132 genes reported for Romney mammary gland by Paten et al. (2015). However, while there were more reads identified in the study than those for a study in Spanish dairy sheep with approximately 30 million reads (Suárez-Vega et al., 2015, 2016a,b, 2017). The number of genes expressed in this study was less than that reported in Spanish dairy sheep by Suárez-Vega et al. (2015). This may be due to the different tissues used to source the RNA. The RNA used for the transcriptome study in Suárez-Vega et al. (2015) was extracted from MSCs, while the RNA used in this study was from the mammary gland parenchyma. In dairy cows, there has been reported to be higher numbers of genes expressed in MSCs than mammary gland tissue (Cánovas et al., 2014).

Of the top ten highly expressed genes found at peak-lactation and in non-lactating sheep, there were two genes (*EEF1A1* and *RPS29*) that were found to be highly expressed in both periods. Of these, EEF1A1 has been described as one of the most abundant protein synthesis factors. It binds amino-acylated tRNAs and facilitate their recruitment to ribosomes during translation elongation (Hershey, 1991). The EEF1A1 gene has been reported to be one of the most stable housekeeping genes identified at different stages of lactation in cows (Pradeep et al., 2014). Ribosomal protein S29 is a structural constituent of ribosome, and it is not that surprising that the RPS29 gene (*RPS29*) was found to be highly expressed in both lactating and non-lactating sheep.

Of the top 10 highly expressed genes during peak-lactation, the most highly expressed genes were the genes encoding for four caseins; β-casein (*CSN2*), α-S1 casein (*CSN1S1*), α-S2 casein (*CSN1S2*), and κ-casein (*CSN3*), and the two whey proteins β-lactalbumin (*LGB*) and α-lactalbumin (*LALBA*). These six genes contributed to approximately 57% of the total gene RPKM values during peaking-lactation, which is higher than reported levels in lactating goats (42%) (Shi et al., 2015). The detection of high levels of expression for these casein and whey protein genes, is reflected in the casein and whey protein content of sheep milk, where together they account for 5.5% of total milk composition (Suárez-Vega et al., 2016b). It is also consistent with other findings for sheep (Paten et al., 2015; Suárez-Vega et al., 2015, 2016b), cows (Wickramasinghe et al., 2012) and humans (Lemay et al., 2013). The β-casein gene (*CSN2*) was found to be the most highly expressed gene in the study, which is in agreement with the observation that β-caseins accounts for 45% of the caseins in milk (Farrell et al., 2004). This is also similar to what has been reported for other species (Lemay et al., 2013; Paten et al., 2015; Crisà et al., 2016; Suárez-Vega et al., 2016a).

There were two genes (*GLYCAM1* and *FASN*) that were also among the top ten expressed genes at peak-lactation, but were hardly expressed in the non-lactating period. The gene *GLYCAM1* encodes a milk fat globule glycoprotein and it is considered to be hormone-regulated protein that is part of the milk-mucin complex (Dowbenko et al., 1993). Fatty acid synthase (FASN) is a rate-limited enzyme for *de novo* fatty acid synthesis during lactation (Bionaz and Loor, 2008), and it is directly involved in the synthesis of most of the short and medium-chain fatty acids in milk. It is considered to be essential for mammary gland development and milk production during lactation (Suburu et al., 2014). The increased expression of both *GLYCAM1* and *FASN* has also been reported in goats (Crisà et al., 2016), and the increased expression of *GLYCAM1* has been reported in cows (Wickramasinghe et al., 2012) and sheep (Paten et al., 2015; Suárez-Vega et al., 2015).

During the lactation, the profile of the top-10 expressed genes found in this study matches well with those reported previously in sheep (Paten et al., 2015) and dairy cattle (Li et al., 2016), except for *RPS29*. The gene *RPS29* was the least expressed gene among the top-10 expressed genes in this study, but is not in the top-10 expressed gene list in the studies of Paten et al. (2015) and Li et al. (2016). The similarity of the top-10 expressed gene lists in this study and those in other studies of lactating sheep and cattle suggests that the RNA-Seq results obtained are reasonable and that sheep and cattle may have the similar mechanisms of mammary gland development and milk synthesis during lactation.

Few transcriptomic studies of mammary gland tissue have been carried out in the non-lactating period of other domestic animals, and hence the most highly expressed genes in the non-lactating mammary gland tissues in this study, could not be compared with previous similar results, such as those described by Dai et al. (2018) and Jiangfeng et al. (2018). Despite there being a study reporting the non-lactating transcriptomic profile of dairy cows (Li et al., 2016), their ranking of expression levels are based on mRNA reads and not the RPKM counts (as used in this study), thus the most expressed genes found in this study and Li et al. (2016), are not easily compared.

In Small-Tailed Han sheep mammary gland tissue it is interesting to observe that *IgG*, *IgA*, and *APOE* are highly expressed in the non-lactating period, but hardly expressed during lactation. The gene *APOE* encodes a fat-binding protein APOE which is a major supplier of cholesterol precursors for the production of steroid hormones, including ovarian estrogen and progesterone. Recent research has shown that *APOE* is associated with fertility in women (Jasienska et al., 2015). Given that the Small-Tailed Han sheep is a high fertile sheep breed, with average lambing rate per ewe being 280%, it is possible that the high expression of *APOE* in the non-lactating mammary gland tissue of Small-Tailed Han sheep may be related to the high fertility of this breed.

Little study has been undertaken into the expression of immunoglobulins in the mammary gland in sheep, but research in dairy cows has revealed that the concentration of immunoglobulins in mammary secretions varied in different stages of lactation (Sordillo et al., 1987). The concentration of immunoglobulins is low during lactation, but slowly increases during the non-lactating periods and reaches peak concentrations during colostrogenesis (Sordillo et al., 1987). The finding that the expression of *IgA* and *IgG* were highly up-regulated in the sheep mammary gland during the non-lactating period, appears to be consistent with this observation in dairy cows. It suggests that the mammary gland may be more susceptible to pathogen infection during non-lactating, but also may explain why early lactation milk, including colostrum, is rich in immunoglobulin and a source of passive immunity for the neonate (Hurley and Theil, 2011).

Overall, the results of this study indicate that in the sheep mammary gland, the development of immune defenses is a hallmark of the non-lactating stage, while a hallmark of peak-lactation is the vast increase in milk protein synthesis. The reduction in the synthesis of other proteins over lactation may enable the majority of available energy to go into the milk synthesis pathway and hence favor milk production.

In sheep mammary gland, the top-10 expressed genes in the peak-lactating period accounted for over 60% of total RPKM values, while the top-10 expressed genes in the non-lactating period accounted for less than 13% of the total RPKM values. This indicates that lactation requires a vast increase in expression of a small number of genes, and a lower level of increase in expression of a wider variety of genes occurs in the non-lactating period. This supports the argument that a greater numbers of genes start to be expressed in the non-lactating period to prepare for the next period of lactation and subsequent parturition.

It is notable that some highly expressed genes were also differentially expressed in the two stages. For example, all the highly expressed genes in the peak-lactation period were significantly down-regulated in the non-lactating period, with the exception of *RPS29*. Of the highly expressed genes during the non-lactation period, *IgG*, *IgA*, and *APOE* were markedly up-regulated. This suggests that the DEGs with high expression levels may play important roles in determining differences in mammary gland development between the non-lactating and peak-lactation periods.

Of the eight down-regulated genes in the non-lactating period validated by RT-qPCR, *XDH*, and *LPL* were also found to be down-regulated in the bovine mammary gland of lactating cows when compared to dry cows (Dai et al., 2018). The two genes are involved in milk fat synthesis and secretion. The protein XDH is one of the main proteins in the milk fat globule membrane and there is a positive association in gene expression between *XDH* and the gene involved in the esterification of fatty acid to glycerol in milk (Bernard et al., 2012). Chylomicrons or very low-density lipoprotein are anchored to mammary endothelium by LPL, which can hydrolysis triacylglycerol in the lipoprotein core to release fatty acid (Fielding and Frayn, 1998). The down-regulation of *STAT5a* and *HSPA9* in the non-lactating mammary gland tissues in this study is likely to be related to their roles in mammary gland development and lactation. The protein STAT5a is necessary for alveogenesis and lactogenesis during lactation and activation of STAT5a can drive side-branching and alveolar differentiation (Haricharan and Li, 2014). The protein HSPA9 plays multiple roles in energy generation, mitochondrial import, intracellular transport and stress responses (Hou et al., 2019). As a member of HSP family, it may also have some more typical HSP functions, and thus play a molecular chaperone role in regulating protein folding and processing, as well as having functions in immunity and inhibition of apoptosis (Paten et al., 2015). Together, this would suggest that *HSPA9* expression may be involved in protein synthesis in ovine mammary gland.

Of the eight up-regulated genes in the non-lactating period validated by RT-qPCR, *STAT6*, *CD4*, and *MAP3K14* are related to immune response. For example, STAT6 is required for T helper cell regulation during immune responses and has also been identified as a regulator of mammary gland differentiation (Haricharan and Li, 2014). The CD4 gene has previously been suggested to have a role as the mammary gland returns to a non-lactating state post-weaning (Betts et al., 2018). In human mammary epithelial cells, *GDF10* has been shown to inhibit proliferation and epithelial mesenchymal transition and induce apoptosis, which is one of typical characteristics in the non-lactating mammary gland (Zhou et al., 2019). In this context, the up-regulation of these genes in the non-lactating period in this study appears to be consistent with what has been described previously.

The GO analysis can help us better understand the function of DEGs and difference in mammary gland development between the two stages. Vesicle was the most enriched GO term with the lowest *P-*value in this study. In mammary gland of lactating ewes, the term was also one of the most enriched categories and it has been revealed to associate with lipid synthesis and secretion (Paten et al., 2015). Consistent with the observations made in this study when comparing lactating and non-lactating mammary gland tissues in sheep, the DEGs in dairy cows have been reported to be enriched in cytoplasm, extracellular region, organelle, membrane, and intracellular functions (Dai et al., 2018) and those in yak were primarily enriched in protein binding (Jiangfeng et al., 2018).

The KEGG of DEGs provided significant insight into the potential biological pathways associated with the mammary gland and lactation in sheep. As the most enriched pathway in the study, ECM-receptor interaction plays pleiotropic roles in mammary gland development, including the regulation of cell adhesion, survival, apoptosis, proliferation and differentiation, organ morphogenesis and tissue structure and function. This pathway has also been described as being related to the onset of mammary gland involution (Maller et al., 2010). Other enriched pathways for the DEGs found in the study, have also been described previously. For example, protein processing in endoplasmic reticulum and aminoacyl-tRNA biosynthesis were enriched by down-regulated genes, and MAPK was primarily enriched by up-regulated genes in the non-lactating mammary gland in dairy cows (Dai et al., 2018). The DEGs of yak mammary glands when comparing between lactation and dry periods have been reported to enrich in protein processing in endoplasmic reticulum, steroid biosynthesis, axon guidance and inflammatory mediator regulation of TRP channels (Jiangfeng et al., 2018). The pathways with majority of DEGs being down-regulated in the non-lactation period in the study, were associated with protein and lipid synthesis, and carbon metabolism.

## Conclusion

The RNA-Seq approach used here to study the mammary gland of lactating and non-lactating sheep produced results that increase our understanding of these states and that are consistent with studies in other species. The development of immune defenses appears to be a hallmark of the non-lactating stage, while a hallmark of peak-lactation is perhaps quite expectedly a large increase in the genes involved in milk protein synthesis.

## Data Availability Statement

The datasets generated for this study can be found in the SRR11300645, SRR11300646, SRR11300647, SRR11300648, SRR11300649, and SRR11300650.

## Ethics Statement

The animal study was reviewed and approved by the Animal Care Committee at Gansu Agricultural University. Written informed consent was obtained from the owners for the participation of their animals in this study.

## Author Contributions

JW, HZ, and JGH: conceptualization. ZH and JS: validation. JH, XL, and SL: investigation. JW, HZ, JGH, and YL: writing. JW and YL: funding acquisition.

## Conflict of Interest

The authors declare that the research was conducted in the absence of any commercial or financial relationships that could be construed as a potential conflict of interest.

## Footnotes

1 http://www.fao.org/resources/infographics/infographics-details/en/c/273893/

2 http://rseqc.sourceforge.net/

3 http://geneontology.org/

4 http://www.genome.jp/kegg/
