# Supplementary material for: Comparison of the Transcriptome of the Ovine Mammary Gland in Lactating and Non-lactating Small-Tailed Han Sheep
Source: Front Genet. 2020 May 21;11:472. doi: 10.3389/fgene.2020.00472 (PMC7253648; doi:10.3389/fgene.2020.00472)
Supplement: Supplementary file 1 [file Data_Sheet_1.DOCX]

**
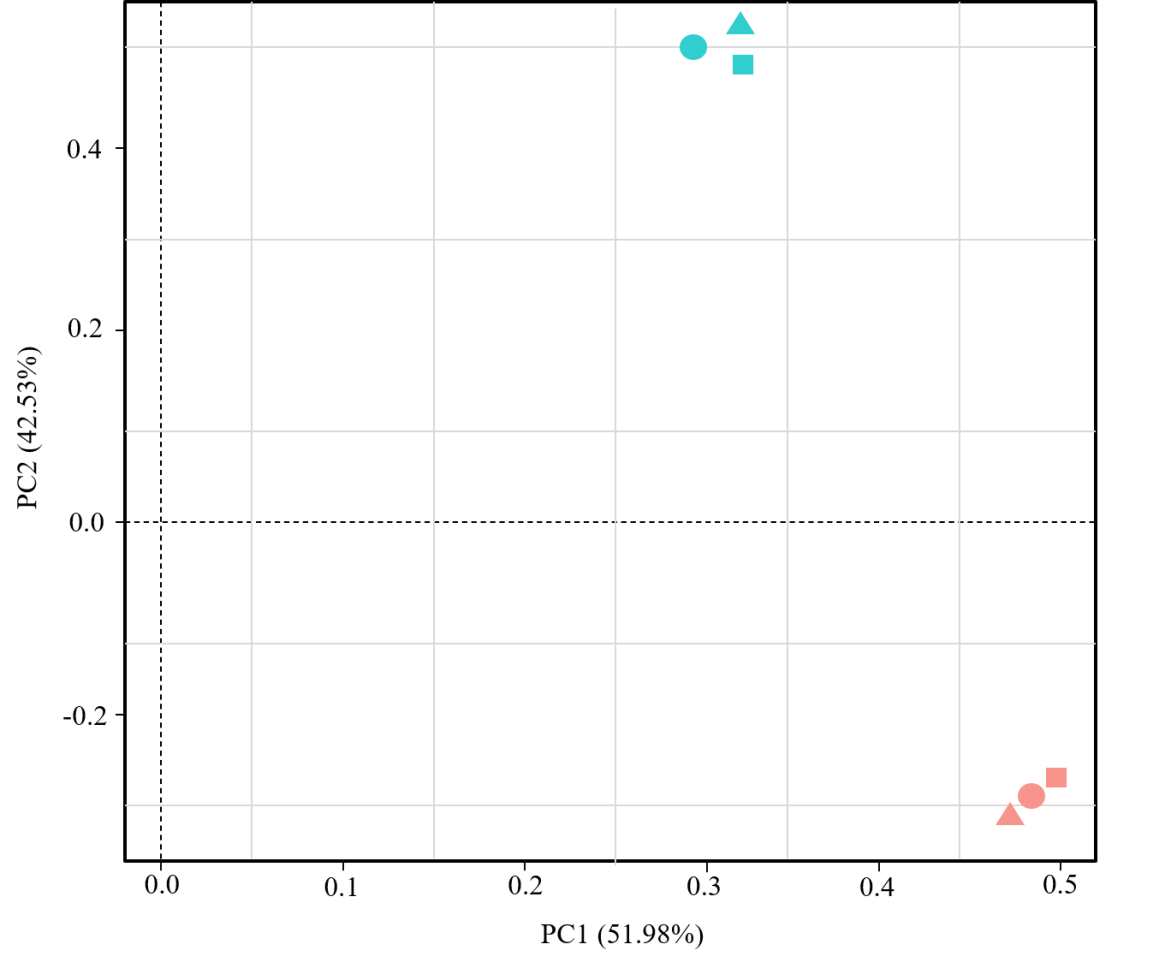
**

**Supplementary file 1.** Principal components analysis (PCA) of gene expression in the ovine mammary gland tissues obtained during the non-lactating (blue) and peak-lactation (red) periods.
