# Supplementary material for: Comparison of the Transcriptome of the Ovine Mammary Gland in Lactating and Non-lactating Small-Tailed Han Sheep
Source: Front Genet. 2020 May 21;11:472. doi: 10.3389/fgene.2020.00472 (PMC7253648; doi:10.3389/fgene.2020.00472)
Supplement: Supplementary file 2 [file Data_Sheet_2.DOCX]

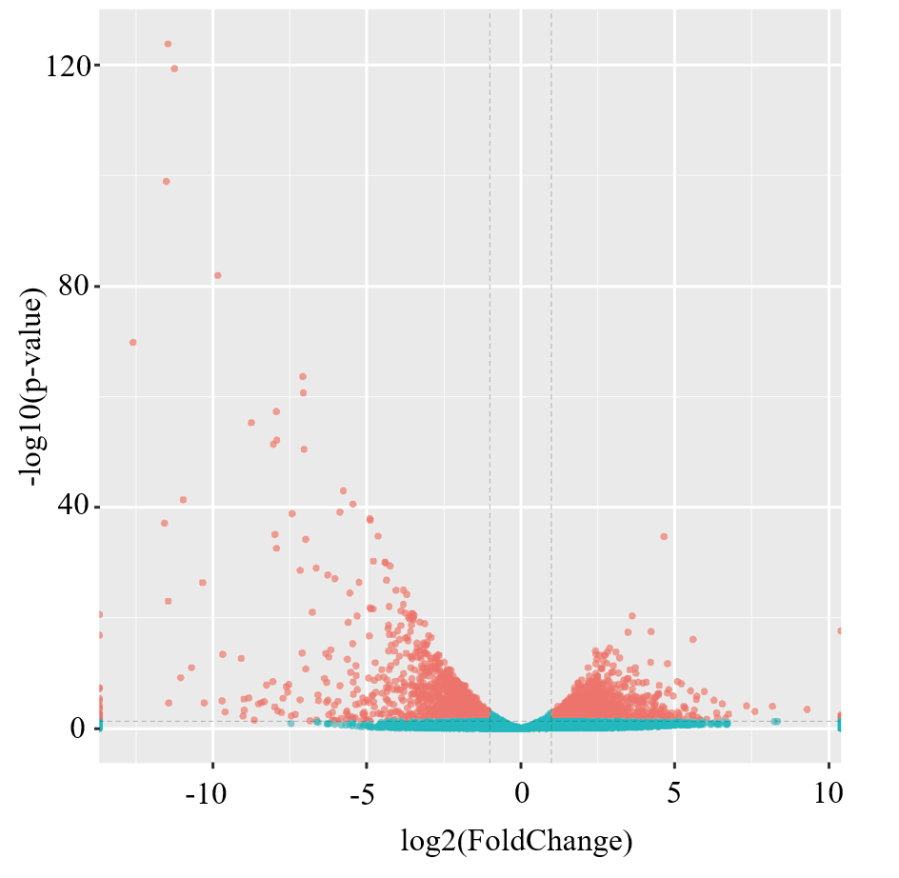


**Supplementary file 2.** Volcano plot showing the change in gene expression of the non-lactating and peak-lactating mammary gland tissues. The red and blue dots represent the differentially expressed genes (*P* < 0.05) and genes that are not significantly different (*P* > 0.05) between the two stages, respectively.
